# Functional analysis of the three *HMA4* copies of the metal hyperaccumulator *Arabidopsis halleri*

**DOI:** 10.1093/jxb/erv280

**Published:** 2015-06-04

**Authors:** Cécile Nouet, Jean-Benoit Charlier, Monique Carnol, Bernard Bosman, Frédéric Farnir, Patrick Motte, Marc Hanikenne

**Affiliations:** ^1^Functional Genomics and Plant Molecular Imaging, Center for Protein Engineering (CIP), Department of Life Sciences, University of Liège, B-4000 Liège, Belgium; ^2^Laboratory of Plant and Microbial Ecology, Department of Biology, Ecology, Evolution, University of Liège, B-4000 Liège, Belgium; ^3^Biostatistics and Bioinformatics, FARAH, Faculty of Veterinary Medicine, University of Liège, B-4000 Liège, Belgium; ^4^PhytoSYSTEMS, University of Liège, B-4000 Liège, Belgium

**Keywords:** Arabidopsis relative, P-type ATPase, cadmium, metal homeostasis, metal hyperaccumulation, metal hypertolerance, zinc.

## Abstract

In *Arabidopsis halleri*, the *HMA4* gene has an essential function in Zn/Cd hypertolerance and hyperaccumulation by mediating root-to-shoot translocation of metals. Constitutive high expression of *AhHMA4* results from a tandem triplication and *cis*-activation of the promoter of all three copies. The three *AhHMA4* copies possess divergent promoter sequences, but highly conserved coding sequences, and display identical expression profiles in the root and shoot vascular system. Here, an *AhHMA4::GFP* fusion was expressed under the control of each of the three *A. halleri HMA4* promoters in a *hma2hma4* double mutant of *A. thaliana* to individually examine the function of each *AhHMA4* copy. The protein showed non-polar localization at the plasma membrane of the root pericycle cells of both *A. thaliana* and *A. halleri*. The expression of each *AhHMA4::GFP* copy complemented the severe Zn-deficiency phenotype of the *hma2hma4* mutant by restoring root-to-shoot translocation of Zn. However, each copy had a different impact on metal homeostasis in the *A. thaliana* genetic background: *AhHMA4* copies 2 and 3 were more highly expressed and provided higher Zn tolerance in roots and accumulation in shoots than copy 1, and *AhHMA4* copy 3 also increased Cd tolerance in roots. These data suggest a certain extent of functional differentiation among the three *A. halleri HMA4* copies, stemming from differences in expression levels rather than in expression profile. HMA4 is a key node of the Zn homeostasis network and small changes in expression level can have a major impact on Zn allocation to root or shoot tissues.

## Highlight

Expression of the *Arabidopsis halleri* hyperaccumulation gene *HMA4* in *A. thaliana* reveals functional differentiation among the three *AhHMA4* copies and non-polar localization of the AhHMA4 protein in root pericycle cells.

## Introduction

Plants possess a complex and tightly regulated metal homeostasis network enabling appropriate metal supply to tissues throughout development (Puig and Peñarrubia, 2009). This allows plants to colonize environments that substantially differ in metal availability, ranging from severe deficiency to toxicity in polluted areas. For instance, so-called metal-hyperaccumulating plants, which are represented by ~500 species, establish populations on soils heavily polluted by metals and are characterized by their capacity to accumulate extremely high concentrations of metals in shoots (e.g. >0.3% of their dry biomass of Zn and 0.01% of Cd) without any toxicity symptoms (Verbruggen *et al.*, 2009; Krämer, 2010; Hanikenne and Nouet, 2011). Metal-hyperaccumulating plants are instrumental in unravelling the molecular and adaptive mechanisms underlying a naturally selected extreme trait (Hanikenne *et al.*, 2008; Verbruggen *et al.*, 2009; Krämer, 2010; Verbruggen *et al.*, 2013) and possibly in the development of technologies for phytoremediation (soil remediation by plants), phytomining (metal extraction from the soil), or biofortification (increasing the amount of essential micronutrients in edible plant parts) (Zhao and McGrath, 2009).

*Arabidopsis halleri* is a model for studying Zn and Cd hyperaccumulation in plants (Clauss and Koch, 2006; Hanikenne *et al.*, 2008; Roosens *et al.*, 2008; Krämer, 2010). *A. halleri* is closely related to the non-accumulator and non-tolerant species *A. thaliana* and *A. lyrata*, with divergence estimated at 3–5.8 and 0.4–2 million years ago, respectively (Yogeeswaran *et al.*, 2005; Clauss and Koch, 2006; Roux *et al.*, 2011). Transcriptomics and quantitative genetics studies allowed the identification of candidate genes for Zn and Cd hypertolerance and hyperaccumulation. These genes contribute to metal homeostasis, namely metal transport, chelation, or detoxification, and are highly expressed in *A. halleri* compared to *A. thaliana* and *A. lyrata* (Becher *et al.*, 2004; Weber *et al.*, 2004; Filatov *et al.*, 2006; Talke *et al.*, 2006; Courbot *et al.*, 2007; Willems *et al.*, 2007; Frérot *et al.*, 2010; Willems *et al.*, 2010).

Among the metal homeostasis genes, *Heavy Metal ATPase 4* (*HMA4*) has been identified as one of the key components contributing to Zn and Cd hyperaccumulation and hypertolerance in *A. halleri* (Talke *et al.*, 2006; Courbot *et al.*, 2007; Willems *et al.*, 2007; Hanikenne *et al.*, 2008; Frérot *et al.*, 2010; Willems *et al.*, 2010). First characterized in *A. thaliana, HMA4* encodes a IB-2 P-type ATPase acting as a Zn/Cd efflux pump (Williams and Mills, 2005; Palmgren and Nissen, 2011; Pedersen *et al.*, 2012; Hanikenne and Baurain, 2014). Together with its paralog HMA2, it is localized at the plasma membrane and is expressed in vascular tissues in roots and shoots (Hussain *et al.*, 2004; Verret *et al.*, 2004; Siemianowski *et al.*, 2013). HMA2 and HMA4 are responsible for the translocation of Zn from roots to shoots: an *hma2hma4* double mutant experiences severe Zn deficiency in shoots and cannot develop normally and set seeds. This phenotype is reversed by massive external Zn supply (Hussain *et al.*, 2004). HMA2 and HMA4 are also responsible for Cd export to the shoots (Wong and Cobbett, 2009).

In *A. halleri*, *HMA4* displays 6- and 30-fold higher transcript levels in roots and shoots, respectively, compared to *A. thaliana* (Talke *et al.*, 2006). The *HMA4* gene co-localizes with major quantitative trait loci for Zn and Cd hyperaccumulation and hypertolerance (Courbot *et al.*, 2007; Willems *et al.*, 2007; Frérot *et al.*, 2010; Willems *et al.*, 2010). High expression of *HMA4* in *A. halleri* is required for high rates of root-to-shoot translocation of Zn by mediating xylem loading in roots and possibly intercellular distribution in leaves (Hanikenne *et al.*, 2008). Increased expression of *HMA4* in *A. halleri* results from tandem triplication and *cis*-regulatory changes activating the promoters of all three *HMA4* copies (Hanikenne *et al.*, 2008; Hanikenne *et al.*, 2013). The promoter of *HMA4* copy 1 (*pAhHMA4-1*) shares ~73% identity with the *AtHMA4* promoter, whereas the promoters of copies 2 and 3 (*pAhHMA4-2* and *pAhHMA4-3*) are highly similar (~82% of identity), but share only limited similarity with the *AtHMA4* promoter (Hanikenne *et al.*, 2008). Each of the three promoters contributes to the elevated expression of *HMA4* and mediate a similar spatial profile of expression in vascular tissues (Hanikenne *et al.*, 2008). Promoter GUS reporter studies established that *HMA4* is expressed in the pericycle and xylem parenchyma in *A. halleri* roots, whereas it is expressed in the xylem parenchyma and cambium in shoots (Hanikenne *et al.*, 2008). An analysis of nucleotide polymorphism patterns at the *AhHMA4* locus provided evidence for positive selection on *cis*-regulatory sequences and/or copy number expansion during the evolutionary history of *A. halleri* (Hanikenne *et al.*, 2013). In addition, coding sequences of the three *AhHMA4* copies are almost identical (>99% nucleotide sequence identity) which results from ectopic gene conversion among gene copies (Hanikenne *et al.*, 2013). Together, this complex nucleotide polymorphism pattern at the *AhHMA4* locus substantiates selection for increased gene product.

*HMA4* is also constitutively more highly expressed in *Noccaea caerulescens*, another Zn and Cd hyperaccumulator species in the Brassicaceae that diverged from *A. thaliana* about 20 million years ago (Verbruggen *et al.*, 2009; Krämer, 2010; Hanikenne and Nouet, 2011). As in *A. halleri*, high expression of *HMA4* in *N. caerulescens* is associated with copy number expansion and regulatory changes (Ò Lochlainn *et al.*, 2011; Craciun *et al.*, 2012), suggesting parallel evolution in the two hyperaccumulator species. Moreover, differences in *HMA4* expression levels between *N. caerulescens* populations exhibiting contrasted metal accumulation and tolerance were associated with gene copy number variations (Craciun *et al.*, 2012).

In this study, genetic constructs were generated to express the *AhHMA4* cDNA in fusion with *GFP* under the control of each of the three *AhHMA4* promoters for transformation in *A. halleri* and in the *hma2hma4* double mutant of *A. thaliana*. This allowed determination of the expression pattern of the HMA4 protein and examination of the contribution of each *AhHMA4* gene copy to Zn/Cd accumulation and tolerance. The reported data suggest functional specialization among *AhHMA4* gene copies.

## Materials and methods

### Plant material, cultivation, and transformation

*A. halleri* (L.) O’Kane and Al-Shehbaz ssp. *halleri* (accession Langelsheim) or *A. thaliana* L. Heynhold (accession Columbia-0, Col-0) and the *A. thaliana hma2hma4* double mutant (Col-0 background, described in Hussain *et al.*, 2004) were used in all experiments. For physiological experiments, *A. thaliana* plants were cultivated in liquid or on solid modified Hoagland medium supplemented with 0.8% (w/v) agar (Agar type M; Sigma-Aldrich) in plastic Petri dishes as previously described (Talke *et al.*, 2006; Hanikenne *et al.*, 2008), either under a photoperiod of 16h (long days) or 8h (short days) light (100 µmol photon m^−2^ s^−1^) in a climate-controlled growth chamber at 21/19°C (day/night).

Before transformation, the *hma2hma4* mutant was cultivated on soil supplied daily with 1mM ZnSO_4_.7H_2_O solution for 7 weeks and then 3mM ZnSO_4_.7H_2_O for 5 weeks. The *hma2hma4* mutant was transformed by floral dip (Clough and Bent, 1998). Homozygous lines were obtained after selection on hygromycin B (20 µg/ml) on half-strength Murashige and Skoog solid medium (Duchefa Biochimie) supplemented with 1% sucrose. For the phenotyping on soil, seeds of the complemented lines, wild-type, and *hma2hma4* mutant were germinated on half-strength Murashige and Skoog solid medium supplemented with 1% sucrose in long days. Then, 18-day-old seedlings were transferred in soil watered with tap water and grown in long days for 6 weeks.

*Agrobacterium tumefaciens*-mediated (GV3101, pMP90) stable transformation of *A. halleri* was performed using a tissue-culture based procedure (Hanikenne *et al.*, 2008).

### Generation of the *pAhHMA4-x::HMA4::GFP* construct

The *pAhHMA4-x::HMA4::GFP* constructs for transformation of the *hma2hma4* mutant and *A. halleri* were generated as follows: (i) the eGFP gene was amplified using primers 5′-tata*gtcgac*atggtgagcaagggcgaggag-3′ containing a *Sal*I restriction site (italic) and 5′-tc*ttaattaa*ttacttgtacagctcgtccatgccgagagtgat-3′ containing a *Pac*I restriction site (italic). The full-length *AhHMA4* cDNA was amplified from an *A. halleri* cDNA library using primers 5′-tata*gtcgac*agcactcacatggtgatggtgg-3′ containing a *Sal*I restriction site (italic) and 5′-caccccgaaaat*ggcgtc*acaaaacaaag-3′ containing a *Bsa*HI restriction site (italic). The promoter fragment and cDNA were fused by ligation at the *Sal*I site. Then the *AhHMA4::GFP* fragment was cloned into the *Bsa*HI/*Pac*I sites of the pBluescript II KS+ vector carrying the promoter of *AhHMA4* copy 1 (*pAhHMA4-1*) (2296bp) as described in Hanikenne *et al.* (2008). (ii) The *pAhHMA4-2* (2284bp) and *pAhHMA4-3* (2030bp) promoters were amplified from *A. halleri* genomic DNA (Langelsheim Lan-3.1; Talke *et al.*, 2006; Hanikenne *et al.*, 2008) using primers 5′-atat*gtcgac*tttctcttcttctttgttttgtgacgcc-3′ containing a *Sal*I restriction site (italic) and 5′-atat*gaattc* **ggcgcgcc**gctctctatcctcctttgtaagttcacc-3′ containing *Sal*I (italic) and *Asc*I (bold) restriction sites and cloned by replacing the *pAhHMA4-1* into the *Bsa*HI/*Asc*I sites of the pBluescript II KS+ vector carrying *AhHMA4::GFP*. (iii) The *pAhHMA4::AhHMA4::GFP* cassettes were *Asc*I/*Pac*I-excised from pBluescript II KS+ and cloned into the corresponding sites of the pMDC32 binary vector (Curtis and Grossniklaus, 2003) from which the 35S promoter had been removed using *Apa*I and *Hind*III. All constructs were verified by sequencing.

### Fluorescence confocal microscopy

The roots of 18-day-old seedlings of three independent complemented *A. thaliana* lines and *A. halleri* lines expressing *pAhHMA4::AhHMA4::GFP* were analysed for each of the three promoters. Images were collected using a SP2 confocal microscope (Leica, Mannheim, Germany) as previously described (Tillemans *et al.*, 2006; Rausin *et al.*, 2010). An Argon/Ion laser (488nm) for excitation of the GFP protein and a Helium/Neon laser (543nm) for excitation of propidium iodide (cell walls) were used. The emission light was dispersed and recorded at 500–540nm for GFP and 600–700nm for propidium iodide. Plasmolysis of the root cells was performed by incubating roots in a 4% (w/v) NaCl solution.

### Tolerance assays

Col-0 (wild-type) and *hma2hma4* mutant plants as well as the complemented mutants (four to five independent T3 homozygous lines per promoter construct) were analysed. The seedlings were cultivated in short days on solid Hoagland medium containing 1 µM ZnSO_4_ for 10 days and then transferred on solid Hoagland medium containing either 1 µM ZnSO_4,_ 150 µM ZnSO_4_, or 40 µM CdSO_4_ (Hanikenne *et al.*, 2008). The root growth was monitored every 2 days and was measured on day 7.

### Analysis of metal accumulation in plants

Col-0 (wild-type) and *hma2hma4* mutant plants as well as the complemented mutants (four to five independent T3 homozygous lines per promoter construct) were analysed. Eighteen days after germination on modified Hoagland medium in short days, the seedlings were transferred to hydroponic trays (Araponics, Tocquin *et al.*, 2003) with Hoagland medium containing 1 μM ZnSO_4_ and grown in short days for 2 weeks before initiating the treatments. Plants were then cultivated on either 0.2 μM ZnSO_4_ or 0.05 μM CdSO_4_ for 4 weeks under long days. Root and rosette tissues of two to three plants were harvested separately and desorbed as described (Talke *et al.*, 2006). Plant tissues were dried at 60°C for 3 days. Tissues (10–50mg) were then acid-digested in a DigiPrep tube with 3ml 65% HNO_3_ (Sigma-Aldrich) on a DigiPrep Graphite Block Digestion System (SCP Science) as follows: 15min at 45°C, 15min at 65°C, and 90min at 105°C. After cooling, sample volumes were adjusted to 10ml with milliQ water, and 200 µl >65% HNO_3_ was added. Metal concentration was measured using inductively coupled plasma atomic emission spectroscopy with a Vista-AX instrument (Varian, Melbourne, Australia).

### Analysis of gene expression in *A. thaliana* plants

Col-0 (wild-type) and *hma2hma4* mutant plants as well as the complemented mutants (three to four independent T3 homozygous lines per promoter construct) were analysed. Eighteen days after germination on modified Hoagland medium in short days, seedlings were transferred to hydroponic trays with Hoagland medium containing 0.2 μM ZnSO_4_ and grown for 5 weeks in short days. Root and rosette tissues were harvested separately from two to four plants per genotype. Total DNase-treated RNA was extracted with RNeasy Plant Mini Kit and DNase set (Qiagen). Quality and quantity of RNA was checked visually by denaturing gel electrophoresis and by photometric analysis (A_260_ and A_280_). Syntheses of cDNA were performed with 500ng of total RNA using Oligo(dT) and the RevertAid H Minus First Strand cDNA Synthesis Kit (Fisher Scientific). Quantitative PCR reactions were performed in 384-well plates with an ABI Prism 7900HT system (Applied Biosystems) using Mesa Green qPCR MasterMix (Eurogentec). A total of three technical repeats were run for each combination of cDNA and primer pair (Supplementary Table S1). Equal amounts of cDNA, corresponding to approximately 6ng of total RNA, were used in each reaction. In addition, each reaction contained 5 µl of Mesa Green qPCR MasterMix and 2.5 pmol of forward and reverse primers in a total volume of 10 µl. The following standard thermal profile was used: 2min at 50°C, 10min at 95°C, 40 repeats of 15 s at 95°C and 60 s at 60°C, and a final stage of 15 s at 95°C, 15 s at 60°C, and 15 s at 95°C to determine dissociation curves of the amplified products. The quality of the PCRs was checked visually through analysis of dissociation and amplification curves, and reaction efficiencies were determined for each PCR using the LinRegPCR software v2013 (Ruijter *et al.*, 2009). Mean reaction efficiencies were then determined for each primer pair from all reactions (>100 reactions; Supplementary Table S1) and used to calculate relative gene expression levels by normalization using multiple reference genes with the qBase software (Biogazelle; Hellemans *et al.*, 2007). Two reference genes (*UBQ10*, *EF1a*) were selected from the literature (Czechowski *et al.*, 2005). Their adequacy to normalize gene expression in the experimental conditions was verified using the geNorm software in qBase (gene stability measure M = 0.464, pairwise variation CV = 0.161) (Vandesompele *et al.*, 2002).

### Statistical analysis

All data evaluation and statistics were done using GraphPad Prism 5 (GraphPad Software) and the GLM procedure (two-way ANOVA) in SAS 9.3 (SAS Software).

### Accession numbers

*A. halleri* sequences are available through EBI (http://www.ebi.ac.uk), accession numbers EU382072, EU382073.

## Results

### Expression of the three *AhHMA4::GFP* copies complemented the *hma2hma4* phenotype

Based on sequence similarity, it was postulated that if functional differences exist among the three *AhHMA4* copies, they are most likely determined by the promoters. To individually examine the function of each *AhHMA4* gene copy, homozygous transgenic lines (T3 generation) were generated of the *hma2hma4* loss-of-function *A. thaliana* mutant expressing the same *AhHMA4* cDNA under the control of each of the three *AhHMA4* promoters. Owing to technical constraints, relatively short versions of the *pAhHMA4-2* and *pAhHMA4-3* promoters (about 1000bp upstream of the ATG translation initiation codon) were cloned previously for a promoter GUS reporter study (Hanikenne *et al.*, 2008). Here, longer DNA fragments of 2284 and 2030bp upstream of the ATG start codon were cloned for *pAhHMA4-2* and *pAhHMA4-3*, respectively, based on available *A. halleri* BAC sequences in the *HMA4* locus (Hanikenne *et al.*, 2008). For the *pAhHMA4-1* promoter, the 2296bp fragment described in Hanikenne *et al.* (2008) was used. Because no antibody raised against the HMA4 protein is available, an *AhHMA4::GFP* fusion was cloned in order to localize the HMA4 protein *in planta*.

**Fig. 1.**
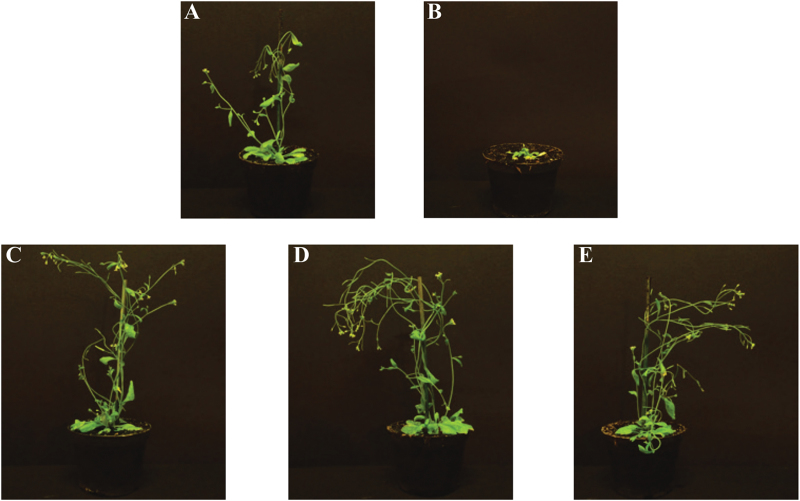
The *hma2hma4* Zn deficiency phenotype can be rescued by *AhHMA4::GFP*. **(A)** Wild-type, and **(B)** *hma2hma4* mutant *A. thaliana* plants as well as transgenic homozygous plants expressing *AhHMA4::GFP* under the control of **(C)** *pAhHMA4-1*, **(D)** *pAhHMA4-2*, and **(E)** *pAhHMA4-3* were grown on soil watered with tap water (this figure is available in colour at *JXB* online).

**Fig. 2.**
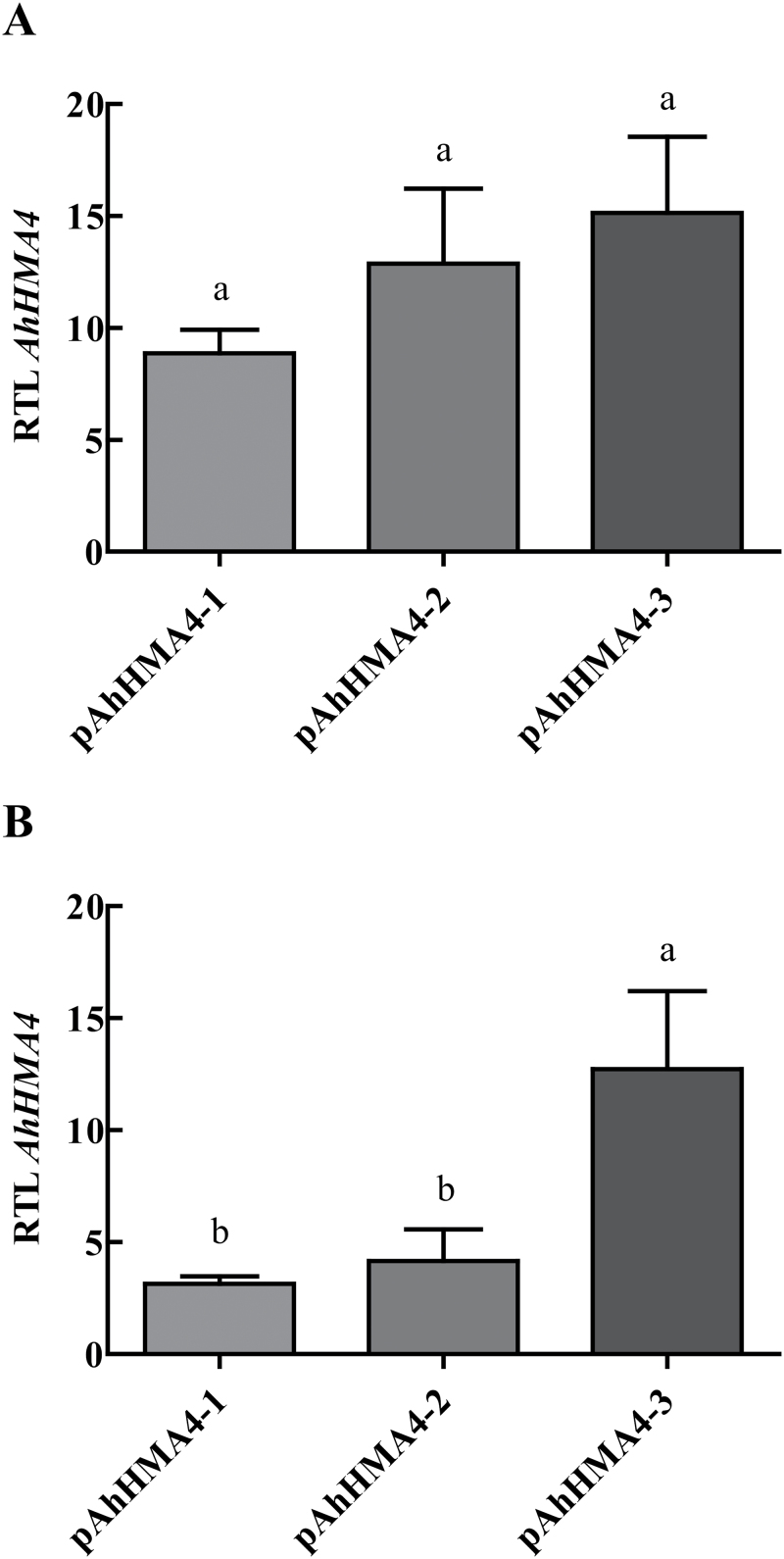
Expression of *AhHMA4* in roots and shoots of the complemented lines. Transgenic homozygous plants expressing *AhHMA4::GFP* under the control of *pAhHMA4-1*, *pAhHMA4-2*, and *pAhHMA4-3* were grown hydroponically with 0.2 µM Zn. Relative expression levels of *AhHMA4* in **(A)** roots and **(B)** shoots are means±SEM of three to four independent lines from one experiment, representative of two independent biological experiments, each including two to four plants per line. The data were analysed with an unpaired *t*-test. Statistically significant differences (*P* < 0.05) between means are indicated by different superscripted letters. RTL: relative transcript level.

In the described growth conditions on standard soil, the *hma2hma4* mutant developed its typical phenotype: stems were very small, which led to a bushy aspect with chlorotic leaves and inflorescences presented no pollen and sterile flowers, in contrast to the wild type (Fig. 1A, B), as previously described (Hussain *et al.*, 2004; Wong and Cobbett, 2009; Mills *et al.*, 2010). Expression of *AhHMA4::GFP* under the control of any of the three *AhHMA4* promoters fully complemented the mutant, restoring a wild-type-like phenotype: the plants developed normally and were able to flower and set seeds without additional Zn supply in the soil (Fig. 1C–E). *HMA4* transcript levels were determined in root and shoot tissues of these plants by quantitative RT-PCR analysis. As expected, *AtHMA4* transcripts were detected in wild-type plants only (Supplementary Fig. S1). *AhHMA4* transcripts were detected consistently in the complemented mutants only and the steady-state levels were higher in roots than in shoots. In roots, *AhHMA4* transcript levels were not significantly different between constructs (Fig. 2A). In contrast, in shoots, the *AhHMA4* transcript levels were 3-fold higher in *pAhHMA-3* lines than in *pAhHMA4-1* and *pAhHMA4-2* lines (Fig. 2B).

### The AhHMA4 protein showed non-polar localization in pericycle cells in roots

**Fig. 3.**
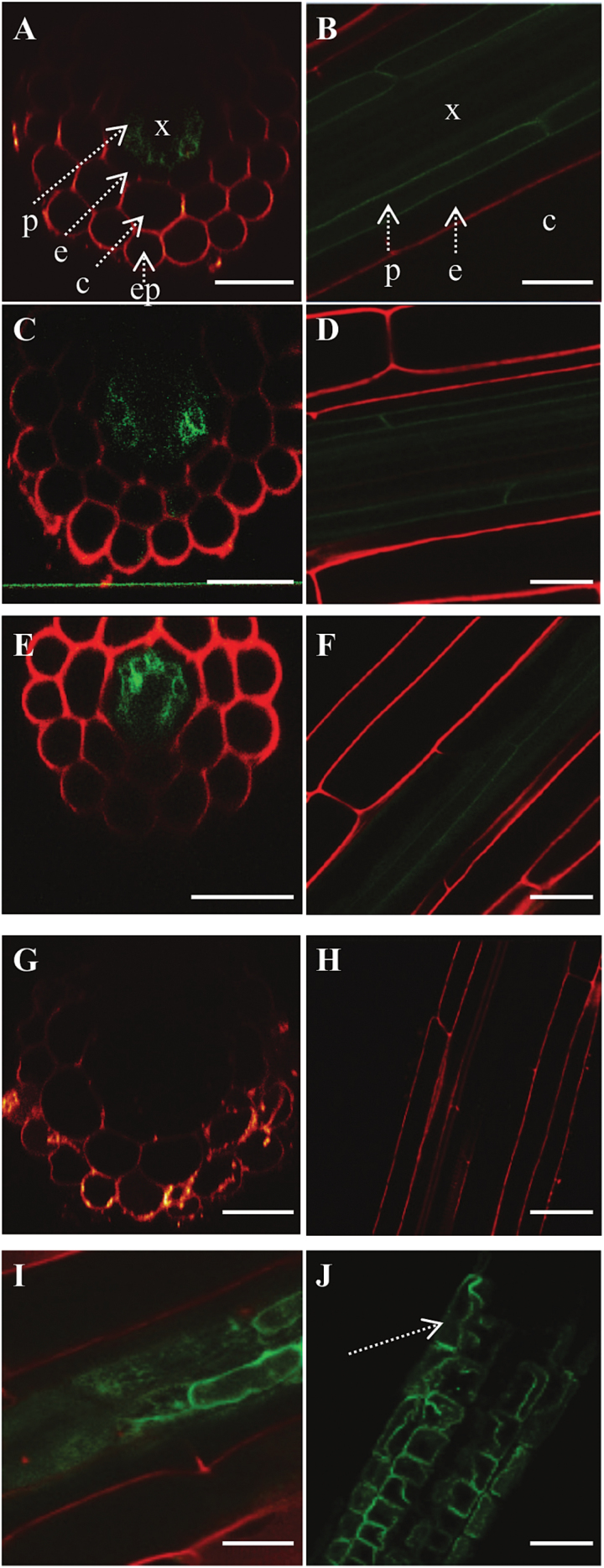
Localization of AhHMA4::GFP fusion protein in *A. thaliana*. GFP fluorescence was imaged by confocal microscopy in roots of 18-day-old seedlings of homozygous *hma2hma4* mutants expressing *AhHMA4::GFP* under the control of **(A, B)** *pAhHMA4-1*, **(C, D)** *pAhHMA4-2*, and **(E, F)** *pAhHMA4-3* promoters. **(G, H)** Wild-type *A. thaliana* seedlings used as control. **(I, J)** Plasmolysis was performed on a *pAhHMA4-1* line, resulting in shrinkage of the plasma membrane. Propidium iodide was used to stain the cell walls (red). Images are representative of a minimum of three independent lines. Dotted arrows identify the different root tissues: c, cortex; e, endodermis; ep, epidermis; p, pericycle; x, xylem. Cross sections: A, C, E, G; longitudinal sections: B, D, F, H, I, J. Scale bars: 30 µm (A, C, E, G, H, J), 20 μm (B, D, F) and 10 µm (I).

Because the AhHMA4::GFP fusion protein was functional (Fig. 1), the complemented mutant transgenic lines were used to examine if each of the three *AhHMA4* promoters determined an identical expression profile at the protein level (Fig. 3). In all cases, the AhHMA4::GFP protein was expressed in the pericycle in roots. The protein localized in the plasma membrane of cells in a non-polar fashion. Performing a plasmolysis of the root cells confirmed the plasma membrane localization (Fig. 3I, J).

**Fig. 4.**
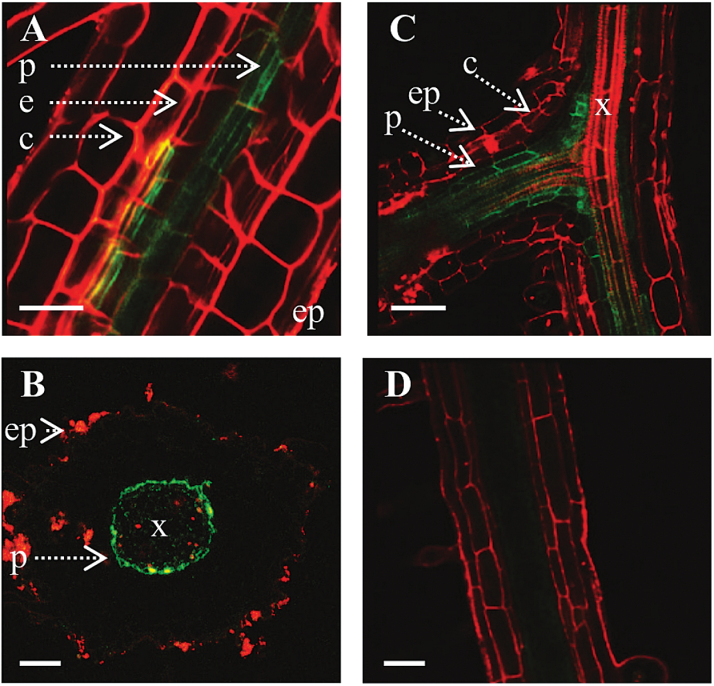
Localization of AhHMA4::GFP fusion protein in *A. halleri*. GFP fluorescence was imaged by confocal microscopy in roots of *A. halleri* expressing *AhHMA4::GFP* under the control of **(A, B)** *pAhHMA4-1* and **(C)** *pAhHMA4-3* promoters and of **(D)** wild-type *A. halleri* as control. Propidium iodide was used to stain the cell walls (red). Images are representative of a minimum of two independent lines. Dotted arrows identify the different root tissues: c, cortex; e, endodermis; ep, epidermis; p, pericycle; x, xylem. Cross sections: B; longitudinal sections: A, C, D. Scale bars: 30 µm (A, B, D) and 50 μm (C).

Localization of AhHMA4::GFP in *A. halleri* transgenics was also studied. Because transforming *A. halleri* is a long and complex process, only *A. halleri* lines expressing *AhHMA4::GFP* under the control of *pAhHMA4-1* and *pAhHMA4-3* could be generated as representatives of the two extreme modifications of the *HMA4* promoter compared to *A. thaliana* (Fig. 4). For these two promoters, the expression profile and sub-cellular localization was identical to that observed in *A. thaliana*. It can be reasonably assumed that *pAhHMA4-2* is also active in the pericycle of *A. halleri* roots. In addition, GFP was observed in cells within the vascular bundle (Fig. 4B).

### Differential Zn and Cd tolerance in complemented lines

**Fig. 5.**
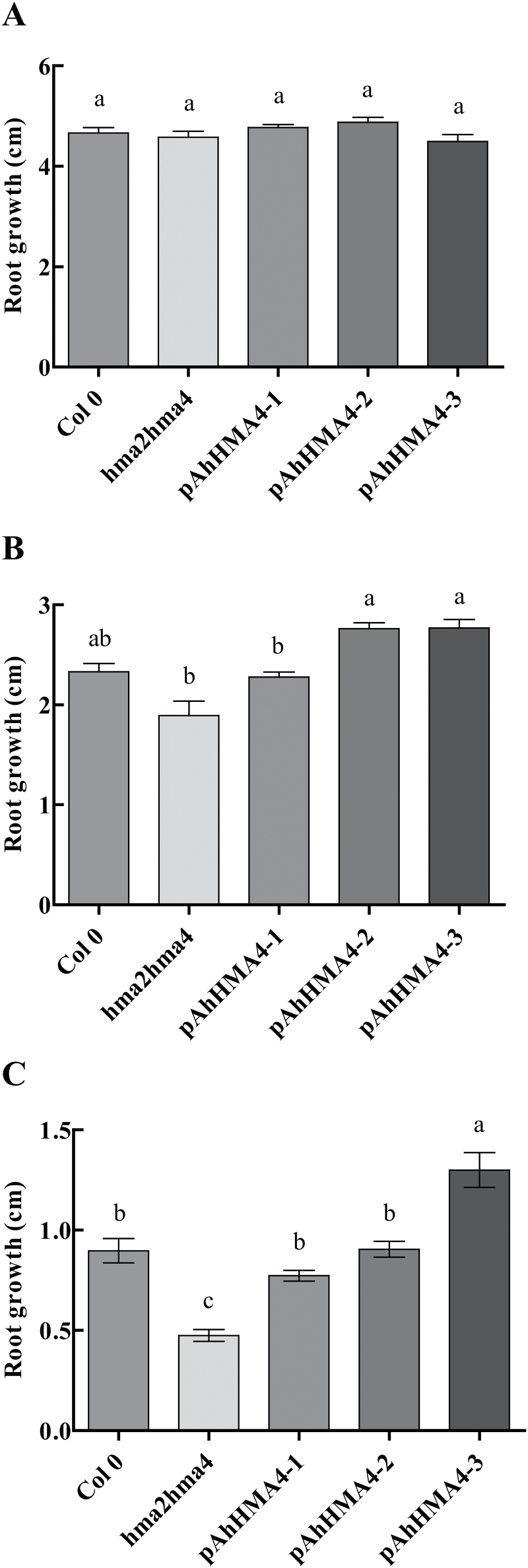
Zn and Cd tolerance in *A. thaliana* complemented lines. Wild-type and *hma2hma4* mutant *A. thaliana* seedlings as well as transgenic homozygous seedlings expressing *AhHMA4::GFP* under the control of *pAhHMA4-1*, *pAhHMA4-2*, and *pAhHMA4-3* were exposed to **(A)** 1 µM ZnSO_4_ (control), **(B)** 150 µM ZnSO_4_, and **(C)** 40 µM CdSO_4_. Root growth values (in cm) are means±SEM and are representative of one experiment out of a total of three independent biological experiments, each including 15–20 seedlings per line/treatment and four to five independent lines. The data were analysed with a two-way ANOVA test with log-transformed values followed by Tukey and Kramer’s multiple comparison tests (*P* < 0.05). Statistically significant differences between means within treatments are indicated by different superscripted letters.

To determine how the expression of *AhHMA4* under the control of each *AhHMA4* promoter contributed to Zn and Cd tolerance, root growth was measured in 10-day-old seedlings (wild-type, *hma2hma4* mutant, and complemented lines) exposed for 7 days on agar medium plates to the control condition (1 µM Zn), Zn excess (150 µM), and Cd (40 µM)_._ In control conditions, the root growth was identical for all genotypes (Fig. 5A). Upon exposure to excess Zn, the root growth of all genotypes was reduced but the *hma2hma4* mutant was more affected, with a 59% growth reduction compared to control conditions (Fig. 5B). The root growths of wild-type and *pAhHMA4-1* lines were similar and ~16% higher than *hma2hma4*. *pAhHMA4-2* and *pAhHMA4-3* lines were the most tolerant with a ~39% higher root growth compared to the double mutant (Fig. 5B). In the presence of Cd, the root growth drastically decreased for all genotypes, by about 70–90% compared to control conditions (Fig. 5C). Again, the *hma2hma4* mutant was the most affected and its growth almost stopped (90% growth reduction compared to control conditions). The root growths of wild-type, *pAhHMA4-1*, and *pAhHMA4-2* lines were similar and 62–90% higher than for *hma2hma4*. The root growth of *pAhHMA4-3* lines was even higher: 173% higher than the double mutant (Fig. 5C).

### Differential Zn and Cd accumulation in complemented lines

**Fig. 6.**
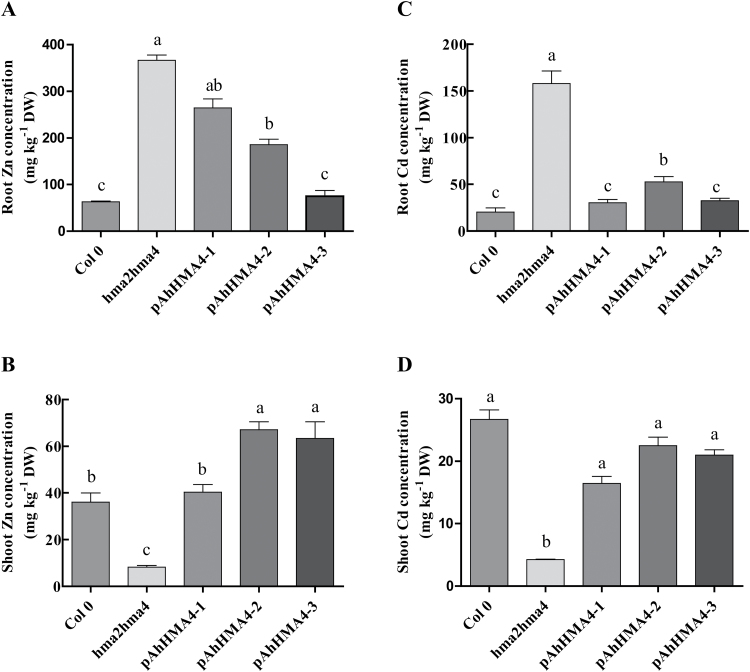
Zn and Cd accumulation in *A. thaliana* complemented lines. Wild-type and *hma2hma4* mutant *A. thaliana* plants as well as transgenic homozygous plants expressing *AhHMA4::GFP* under the control of *pAhHMA4-1*, *pAhHMA4-2*, and *pAhHMA4-3* were cultivated hydroponically with **(A, B)** 0.2 µM ZnSO_4_ and **(C, D)** 0.05 µM CdSO_4_/1 µM ZnSO_4_. Zn and Cd concentrations (mg kg^−1^ dry weight) were measured in root and rosette tissues collected from two to three plants per line. Values are means±SEM of four to five independent lines from one experiment and are representative of three independent experiments. The data were analysed with a two-way ANOVA test with log-transformed values followed by Tukey and Kramer’s multiple comparison tests (*P* < 0.05). Statistically significant differences (*P* < 0.05) between means within each figure panel are indicated by different superscripted letters.

How the expression of *AhHMA4* under the control of each *AhHMA4* promoter contributed to metal accumulation was also determined. Zn and Cd concentrations were measured in the roots and rosette leaves of wild-type, *hma2hma4* mutant, and complemented plants after cultivation for 4 weeks in Hoagland hydroponic medium containing either 0.2 µM Zn or 0.05 µM Cd. Hoagland medium classically contains 1 µM Zn (Becher *et al.*, 2004; Talke *et al.*, 2006), and at this Zn concentration, the growth of the *hma2hma4* mutant was rescued: it could flower and set seeds similarly to the wild type (data not shown). A concentration of 0.2 µM Zn was optimal to distinguish the mutant from the wild type in these growth conditions and assess complementation by the *AhHMA4::GFP* constructs. Indeed, at 0.2 µM Zn, the *hma2hma4* mutant displayed a Zn-deficiency phenotype, whereas all complemented lines displayed a wild-type phenotype (Supplementary Fig. S2). The *hma2hma4* mutant accumulated about 6-fold higher Zn in roots and 4.4-fold lower Zn in shoots than the wild type, respectively (Fig. 6A, B). In roots, the complemented lines accumulated less Zn than the mutant: root Zn accumulation was intermediate between wild type and mutant in *pAhHMA-1* and *pAhHMA-2* lines while the *pAhHMA-3* lines accumulated the smallest Zn amount, at a level similar to the wild type (Fig. 6A). In contrast, Zn accumulation in shoots was 4.4-fold higher in the *pAhHMA-1* lines than in the *hma2hma4* mutant and similar to the wild type. The *pAhHMA-2* and *pAhHMA-3* lines accumulated even more Zn: ~8-fold more than the *hma2hma4* mutant and 1.8-fold more than the wild type (Fig. 6B). Identical observations were made upon 0.05 µM Cd exposure (in the presence of 1 µM Zn): expression of AhHMA4::GFP in the *hma2hma4* mutant restored Cd accumulation in shoots and, in *pAhHMA-2* and *pAhHMA-3* lines, the Cd levels were similar to wild-type levels in roots and shoots (Fig. 6C, D).

### Differential expression of Zn status-responsive genes in complemented lines

**Fig. 7.**
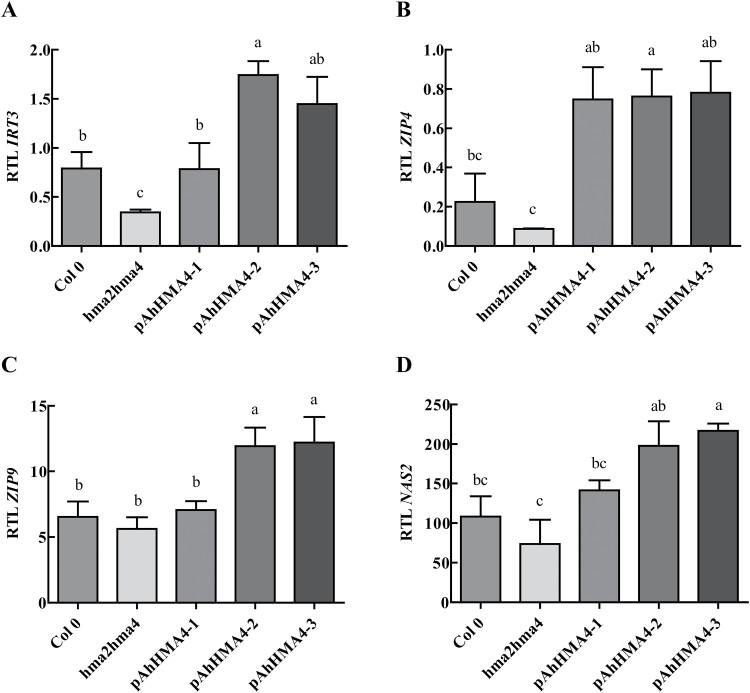
Expression of Zn-responsive genes in roots of *A. thaliana* complemented lines. Wild-type and *hma2hma4* mutant *A. thaliana* plants as well as transgenic homozygous plants expressing *AhHMA4::GFP* under the control of *pAhHMA4-1*, *pAhHMA4-2*, and *pAhHMA4-3* were grown hydroponically with 0.2 µM Zn. Relative expression levels of **(A)** *IRT3*, **(B)** *ZIP4*, **(C)** *ZIP9*, and **(D)** *NAS2* are presented as means±SEM of three to four independent lines from one experiment representative of two independent biological experiments, each including two to four plants per line. The data were analysed with an unpaired *t*-test. Statistically significant differences (*P* < 0.05) between means are indicated by different superscripted letters. RTL: relative transcript level.

To further examine how the expression of *AhHMA4* under the control of each *AhHMA4* promoter contributed to Zn translocation from roots to shoots, the expression level of four genes that respond to Zn status in tissues was measured: *ZIP4, ZIP9*, and *IRT3* encoding Zn uptake transporters, and *NAS2* encoding an enzyme involved in the synthesis of the Zn chelator nicotianamine (NA). The four genes are up-regulated by Zn deficiency and down-regulated by Zn excess in *A. thaliana* (Talke *et al.*, 2006). Gene expression in roots and shoots of plants hydroponically cultivated at 0.2 µM Zn for 5 weeks was analysed by quantitative RT-PCR. In roots, for all four genes, the steady-state transcript levels were lower (up to 75%) in the *hma2hma4* mutant than in the wild type (Fig. 7), as expected: the increased Zn accumulation in roots of the mutant (Fig. 6A) resulted in a down-regulation of the genes. In roots, *ZIP4* steady-state transcript levels were higher in all complemented lines than in the wild type (3.4-fold) and the *hma2hma4* mutant (8-fold) (Fig. 7B). In addition, the steady-state transcript levels of *IRT3, ZIP9*, and *NAS2*, were 2- to 5.4-fold higher in the *pAhHMA4-2* and *pAhHMA4-3* lines compared to the *pAhHMA4-1*, wild type, and *hma2hma4* mutant lines (Fig. 7A, C, D), indicative of Zn depletion in root tissues.

**Fig. 8.**
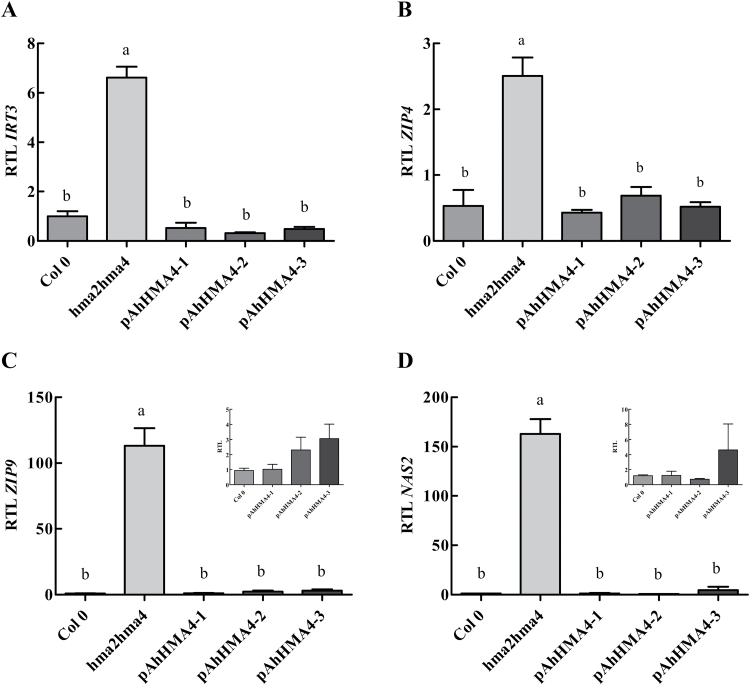
Expression of Zn-responsive genes in shoots of *A. thaliana* complemented lines. Wild-type and *hma2hma4* mutant *A. thaliana* plants as well as transgenic homozygous plants expressing *AhHMA4::GFP* under the control of *pAhHMA4-1*, *pAhHMA4-2*, and *pAhHMA4-3* were grown hydroponically with 0.2 µM Zn. Relative expression levels of **(A)** *IRT3*, **(B)** *ZIP4*, **(C)** *ZIP9*, and **(D)** *NAS2* are presented as means±SEM of three to four independent lines from one experiment representative of two independent biological experiments, each including two to four plants per line. The data were analysed with an unpaired *t*-test. Statistically significant differences (*P* < 0.05) between means are indicated by different superscripted letters. RTL: relative transcript level.

In contrast, in shoots, all genes were more highly expressed in the *hma2hma4* mutant (5 to 137-fold) compared to the wild type (Fig. 8), highlighting the major Zn deficiency in shoots of the mutant (Hussain *et al.*, 2004). Steady-state transcript levels of all genes were massively down-regulated in complemented lines compared to the mutant. The steady-state transcript levels of *IRT3* and *ZIP4* in all complemented lines were similar to the wild type (Fig. 8A, B). Interestingly, the steady-state transcript levels of *ZIP9* and *NAS2* were higher, although not significantly, in shoots of the *pAhHMA4-3* lines than in the other complemented lines (Fig. 8C, D).

## Discussion

So far, no data has been available evaluating separately the contribution of each of the three *AhHMA4* copies to Zn homeostasis. Here, the possible functional differentiation among the three *AhHMA4* copies was examined and the sub-cellular localization and expression profile of the AhHMA4 protein determined when expressed under the control each *AhHMA4* promoter.

### The HMA4 protein is expressed in the root pericycle

The sub-cellular localization of the *A. thaliana* or *A. halleri* HMA4 protein in the plasma membrane has so far only been determined by ectopic over-expression in yeast, *A. thaliana* protoplasts, or tobacco (Verret *et al.*, 2004; Verret *et al.*, 2005; Courbot *et al.*, 2007; Siemianowski *et al.*, 2013). Here, using the *AhHMA4* promoters allowed the determination of the sub-cellular localization of the reporter protein upon tissue-specific expression. When expressed under the control of the *AhHMA4* promoters, the AhHMA4::GFP fusion was functional *in planta* (Fig. 1) and localized in the plasma membrane of pericycle cells in roots of both species (Figs. 3 and 4). As with promoter GUS reporter constructs (Hanikenne *et al.*, 2008), the three *AhHMA4* promoters determined an identical pattern of localization. The AtHMA2 protein, the paralog of AtHMA4, has a similar expression pattern in root cells (Hussain *et al.*, 2004; Sinclair *et al.*, 2007). In *A. halleri*, GFP was also observed in cells within the vascular bundle, which likely correspond to the xylem parenchyma of roots, again consistent with GUS staining (Hanikenne *et al.*, 2008).

Zn hyperaccumulation is a directional process where Zn is very efficiently translocated from the root to the shoot. After cellular uptake in the root epidermis, Zn is transported radially towards the xylem. A polar localization of several membrane transporters has been observed in plant cells, providing directional substrate transport in tissues. For instance, the polar localization of PIN auxin efflux carriers is key for the control of plant growth (Tanaka *et al.*, 2006). In *Oryza sativa* (rice), the Lsi1 and Lsi2 transporters act together to provide polarized influx and efflux of silicon in root exodermal and endodermal cells, respectively (Ma *et al.*, 2006; Ma *et al.*, 2007). Current models representing the function of HMA4 in root cells hypothesize that the protein accumulates on one side of the pericycle cell membrane, directly adjacent to the xylem, allowing Zn loading (Hanikenne *et al.*, 2008; Palmer and Guerinot, 2009; Krämer, 2010; Hanikenne and Nouet, 2011; Claus *et al.*, 2013). In contrast, the HMA4 protein appears to be distributed uniformly, in a non-polar fashion, in the plasma membrane of pericycle cells. The observations presented here suggest that HMA4 contributes to the mobilization and efflux of cellular Zn within the root cells delimited by the Casparian strip. Once in the apoplast, Zn becomes available for xylem loading.

### *AhHMA4* expression in *hma2hma4* complemented lines

In roots of the complemented *hma2hma4* mutant lines, the *AhHMA4* transcript levels were slightly, but not significantly, higher in *pAhHMA4-3* lines compared to *pAhHMA4-1* lines (Fig. 2A). This is consistent with previously published data on GUS activity in promoter GUS reporter lines, where no major differences between promoters were observed (Hanikenne *et al.*, 2008). However, in shoots, *pAhHMA4-3* led to *AhHMA4* steady-state levels 3-fold higher than *pAhHMA4-1* and *pAhHMA4-2*, whereas GUS activity levels were similar for all three promoters (Fig. 2B, Hanikenne *et al.*, 2008). Two hypotheses can be suggested to explain this discrepancy in shoots. First, levels of *AhHMA4::GFP* transcript were measured, whereas the GUS assay measures protein activity. Disconnection between transcript levels and protein levels is not rare (Ghazalpour *et al.*, 2011; Kuersten *et al.*, 2013). These two measurements can thus provide different results as translation efficiency of the transcripts or protein stability can vary (Dvir *et al.*, 2013; Kim *et al.*, 2014; Remy *et al.*, 2014). Note that the AhHMA4::GFP protein fusion could not be detected in western blots to evaluate protein levels. Second, larger fragments for *pAhHMA4-2* (2284bp) and *pAhHMA4-3* (2030bp) were cloned in this study compared to Hanikenne *et al.* (2008). Pairwise comparisons (BLASTN, NCBI) of the three *AhHMA4* promoters used in this study and the *AtHMA4* promoter used in Hanikenne *et al.* (2008) identified six regions with more than 70% identity in the promoters (A–F in Supplementary Fig. S3). This analysis confirmed that promoters *pAhHMA4-2* and *pAhHMA4-3* shared a high level of identity (83%) and diverged substantially from *pAtHMA4* and *pAhHMA-1*. A conserved block (F in Supplementary Fig. S3) is specific to *pAhHMA4-2* and *pAhHMA4-3*. These sequence differences may be responsible for differential *AhHMA4* expression in the complemented lines and may determine the differences in Zn tolerance and accumulation between *pAhHMA4-2*/-*3* and *pAhHMA4-1* lines. Further work will be required to dissect the function of the promoters.

### Expression of the *AhHMA4* complements the *hma2hma4* mutant phenotype

The expression of *AhHMA4::GFP* under the control of each of the three *AhHMA4* promoters complemented the phenotypes of the *hma2hma4* mutant: it restored root-to-shoot translocation of Zn, abolished Zn deficiency in shoots, and enabled the plants to complete their life cycle. The expression of *AhHMA4* also increased Zn and Cd tolerance in roots and accumulation in shoots. This was reflected by changes in the expression levels of Zn-regulated genes, which were back to wild-type levels when compared to the mutant. These genes are up-regulated by Zn deficiency and down-regulated by Zn excess in *A. thaliana* (Talke *et al.*, 2006). This indicated that all three copies of the *AhHMA4* gene are functional in *A. thaliana*. This is in contrast with a recent report by Iqbal *et al.* (2014). In their study, the expression of *A. thaliana* or *N. caerulescens HMA4* cDNAs under the control of *N. caerulescens HMA4* promoters, representing different gene copies from three different accessions of *N. caerulescens*, did not complement the *hma2hma4 A. thaliana* mutant and rather exacerbated the shoot Zn-deficiency phenotype. The aggravation of the phenotype was positively correlated with the *HMA4* expression level. Because the two species are phylogenetically more distant, these observations may be linked to the non-conserved specificity of expression of *N. caerulescens HMA4* promoters in *A. thaliana* tissues, with high expression in leaf mesophyll cells and ectopic expression in roots (Iqbal *et al.*, 2014).

### Copy-specific phenotypes are linked to differences in *HMA4* expression level

If the expression of *AhHMA4::GFP* under the control of each of the three *AhHMA4* promoters complemented the phenotypes of the *hma2hma4* mutant, there was substantial differences among copies. A subset of complemented lines displayed increased root tolerance to Zn (*pAhHMA4-2* and *pAhHMA4-3*) and Cd (*pAhHMA4-3* only) compared to wild-type, mutant, and *pAhHMA4-1* lines.

In a previous study, the expression of *AhHMA4* under the control of *pAhHMA4-1* in Col-0 wild-type plants resulted in a marginal increase (~16%) of Zn accumulation in shoots (Hanikenne *et al.*, 2008). Expression of the same construct in *Nicotiana tabacum* and *Solanum lycopersicum* also resulted in moderate changes in shoot Zn contents, which were dependent on Zn supply in the medium, and in alterations of the metal homeostasis network (Barabasz *et al.*, 2010; Barabasz *et al.*, 2012; Antosiewicz *et al.*, 2014). Here, expression of *AhHMA4::GFP* under the control of *pAhHMA4-1* in the *hma2hma4* double mutant restored wild-type levels of shoot Zn accumulation (Fig. 5). Interestingly, *pAhHMA4-2* and *pAhHMA4-3* transgenic lines displayed ~800% and ~180% increased accumulation of Zn in shoots compared to the mutant and the Col-0 wild type, respectively (Fig. 6B). This represents a substantial increase in shoot accumulation. Indeed, summing the effect of each *HMA4* copy shows that the increase in Zn shoot accumulation compared to the wild type was 4.5-fold, which is in the range of previous comparisons of *A. thaliana* and *A. halleri* grown at low Zn supply (Talke *et al.*, 2006). Note that the *pAhHMA-2* and *pAhHMA-3* promoters also had a higher impact on Cd accumulation than *pAhHMA-1* (Fig. 6D).

The expression of Zn-responsive genes reflected changes in Zn accumulation in the complemented lines tissues. Indeed, *IRT3*, *ZIP4*, *ZIP9*, and *NAS2* steady-state transcript levels in roots were highest in the *pAhHMA4-2* and *pAhHMA4-3* lines compared to the wild type (Fig. 7). This indicated that the *pAhHMA4-2*- and *pAhHMA4-3*-dependent expression of *AhHMA4* resulted in a more efficient Zn translocation from roots to shoots and higher Zn depletion in roots compared to the wild-type, the *hma2hma4* mutant, and the *pAhHMA4-1* lines.

Claus *et al.* (2013) modelled Zn transport in *A. thaliana* roots describing the spatio-temporal evolution of Zn concentration in the symplast and apoplast depending on Zn supply. The model predicted that, in addition to radially oriented advection and the cylindrical geometry of the roots, HMA4 protein level affects the overall Zn concentration in the pericycle, and suggested that a slight change in *AtHMA4* transcript level could drastically modify the Zn gradient from roots to shoots. This is in agreement with the function of *HMA4* in *A. halleri* where increased expression supports an enhanced Zn flux from the root symplasm into the xylem vessels and contributes to hyperaccumulation. The activity of *AhHMA4* in roots determines Zn root to shoot ratios and regulates Zn-responsive gene expression in roots (Hanikenne *et al.*, 2008).

In this context, an examination was performed to determine whether the phenotypes observed in the complemented lines correlated with *AhHMA4* transcript levels. To a certain extent, root tolerance correlated with the level of *AhHMA4* transcripts in roots (Supplementary Fig. S4). Root tolerance to Cd also correlated with *AhHMA4* expression level in shoots, where *AhHMA4* might provide efficient xylem unloading of Cd, acting as a sink to decrease Cd accumulation in roots. In addition, root Zn accumulation negatively correlated with *AhHMA4* expression levels in roots. *AhHMA4* expression also moderately influenced shoot Zn and Cd accumulation (Supplementary Fig. S5). The expression levels of *ZIP9*, *ZIP4*, and *NAS2*, and to a lesser extent *IRT3*, correlated with *AhHMA4* expression in roots, indicating a more efficient transport of Zn to shoots resulting in Zn depletion in roots (Supplementary Fig. S6A-D).

Altogether, these observations suggest that higher expression of *AhHMA4* in roots of *pAhHMA4-2* and *pAhHMA4-3* lines was responsible for the better tolerance and accumulation of Zn, as well as differential expression of Zn-responsive genes.

### Physiological impact of high *AhHMA4* expression in shoots

In shoots of *pAhHMA4-3* lines, the steady-state expression levels of *ZIP9* and *NAS2* were higher than in *pAhHMA4-1* and *pAhHMA4-2* and wild-type lines despite a higher accumulation of Zn in shoot tissues (Fig. 8C, D). Higher accumulation of Zn in shoots should result in decreased expression of these Zn-responsive genes (Talke *et al.*, 2006). Similarly, a negative correlation between *AhHMA4* and Zn-responsive gene expression is expected (Hanikenne *et al.*, 2008). In contrast, a positive correlation between *AhHMA4* expression level and the expression of *ZIP9* and *NAS2* in shoots was observed here (Fig. S6G, H). High expression of *AhHMA4* in *pAhHMA4-3* lines possibly depletes Zn in shoot cells and triggers a Zn-deficiency response in shoots in the presence of high Zn concentration (see also Antosiewicz *et al.*, 2014; Iqbal *et al.*, 2014). As a result, Zn may be mislocalized in the apoplast in shoots of *pAhHMA4-3* lines.

## Conclusion

The data presented here suggest a certain extent of functional differentiation among the three *AhHMA4* copies when expressed in *A. thaliana*, stemming from differences in expression levels rather than in expression profile. *AhHMA4* copies 2 and 3 provide higher Zn tolerance and accumulation than copy 1 (Hanikenne *et al.*, 2008). Interestingly, *AhHMA4* copy 3 was subjected to the strongest, possibly most recent, positive selection during the evolutionary history of *A. halleri* (Hanikenne *et al.*, 2013). The present study thus links evolutive sequence diversity patterns and function *in vivo*. The data further suggest that *AhHMA4* copies 2 and 3 are possibly better targets for phytoremediation and biofortification purposes (Clemens *et al.*, 2002; Palmgren *et al.*, 2008). Stacking up the different copies of *AhHMA4* in *A. thaliana* may further increase tolerance and accumulation, although it may also result in a more pronounced deregulation of Zn homeostasis in shoots (Antosiewicz *et al.*, 2014).

## Supplementary data

Supplementary data are available at *JXB* online.

Supplementary Fig. S1. Expression of *AtHMA4* in roots and shoots of the complemented mutants.

Supplementary Fig. S2. Phenotype of the complemented lines grown in Hoagland hydroponic medium.

Supplementary Fig. S3. Sequence conservation in *HMA4* promoters of *A. thaliana* and *A. halleri*.

Supplementary Fig. S4. Relationship between root growth and *AhHMA4* transcript levels.

Supplementary Fig. S5. Relationship between Zn or Cd accumulation and *AhHMA4* transcript levels.

Supplementary Fig. S6. Relationship between expression of Zn-responsive genes and *AhHMA4*.

Supplementary Table S1. Sequences and reaction efficiencies of primer pairs used for real-time RT-PCR.

Supplementary Data
