# Supplementary material for: Functional analysis of the three HMA4 copies of the metal hyperaccumulator Arabidopsis halleri
Source: J Exp Bot. 2015 Jun 4;66(19):5783–95. doi: 10.1093/jxb/erv280 (PMC4566976; doi:10.1093/jxb/erv280)
Supplement: Supplementary Data [file supp_66_19_5783__index.html]

Functional analysis of the three HMA4 copies of the metal hyperaccumulator Arabidopsis halleri — Functional analysis of the three HMA4 copies of the metal hyperaccumulator Arabidopsis halleri — Functional analysis of the three HMA4 copies of the metal hyperaccumulator Arabidopsis halleri — Supplementary Data 

# Functional analysis of the three *HMA4* copies of the metal hyperaccumulator *Arabidopsis halleri*

## Supplementary Data

Data files

- Supplementary Data - Supplementary Data
