# Supplementary material for: Functional analysis of the three HMA4 copies of the metal hyperaccumulator Arabidopsis halleri
Source: J Exp Bot. 2015 Jun 4;66(19):5783–95. doi: 10.1093/jxb/erv280 (PMC4566976; doi:10.1093/jxb/erv280)
Supplement: Supplementary Data [file supp_erv280_jexbot148320_file001.pdf]

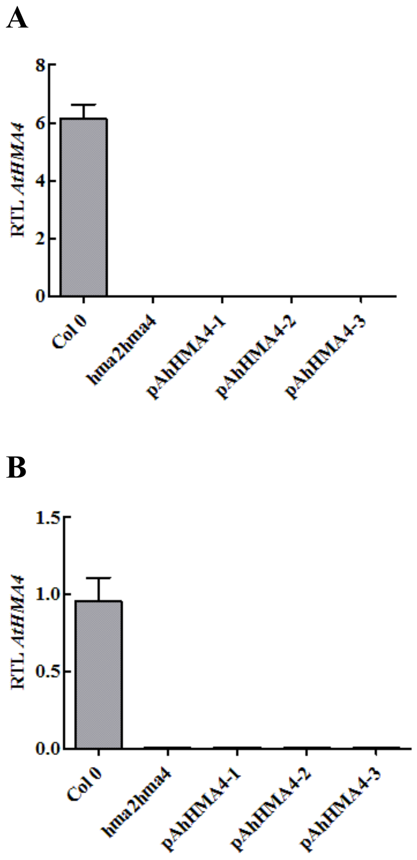

Fig. S1. Expression of *AtHMA4* in roots and shoots of the complemented mutants. 18-days old seedlings of wild-type and *hma2hma4* mutant *A. thaliana* plants as well as transgenic homozygous plants expressing *AhHMA4::GFP* under the control of *pAhHMA4-1*, *pAhHMA4-2* and *pAhHMA4-3* were grown hydroponically in Hoagland medium containing 0.2 µM Zn for five weeks. Expression levels of *AtHMA4* in roots (A) and in shoots (B) were normalized to *EF1α* and *UBQ10*. Values are mean ± SEM of 3-4 independent lines from one experiment representative of two independent biological experiments, each including 2-4 plants/line. RTL: Relative Transcript Level.

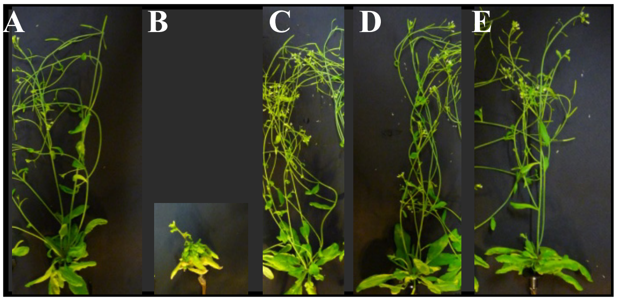

Fig. S2. Phenotype of the complemented lines grown in Hoagland hydroponic medium. Four week-old wild-type (A) and *hma2hma4* mutant *A. thaliana* plants (B) as well as transgenic homozygous plants expressing *AhHMA4::GFP* under the control of *pAhHMA4-1* (C), *pAhHMA4-2* (D) and *pAhHMA4-3* (E) were cultivated hydroponically in Hoagland medium with 0.2 µM $ZnSO_4$ for four weeks.

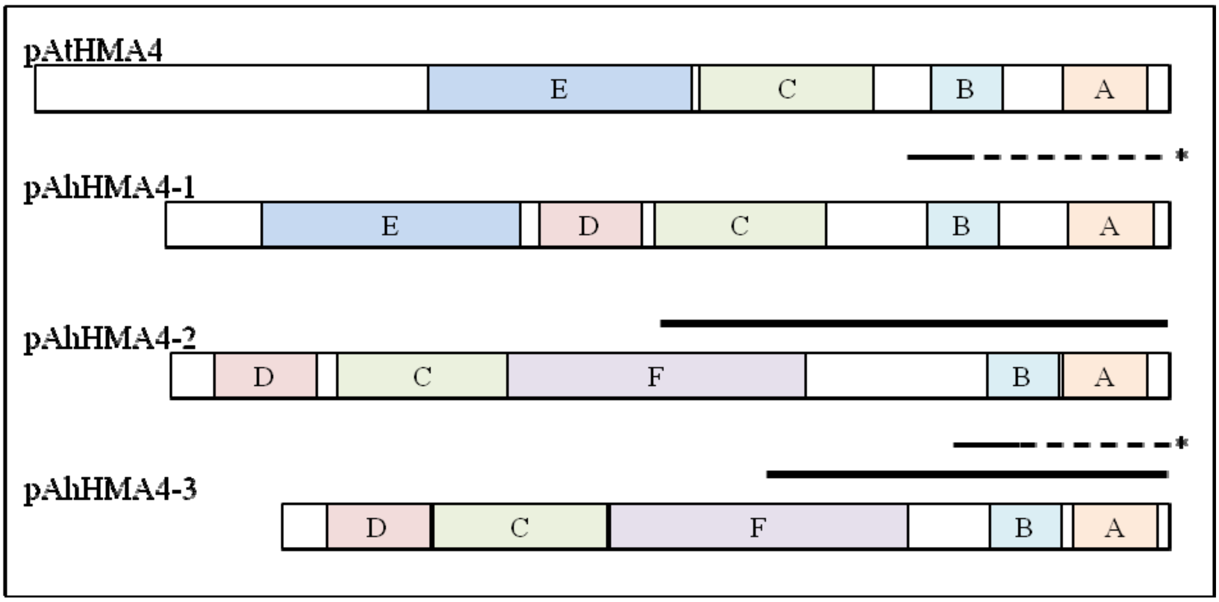

% identity	*pAhHMA4-1*					*pAhHMA4-2*					*pAhHMA4-3*					
	A	*B*	*C*	*D*	*E*	*A*	*B*	*C*	*D*	*E*	*A*	*B*	*C*	*D*	*E*	*F*
pAtHMA4	77	77	94	-	84	75	78	77	-	-	75	78	76	-	-	-
pAhHMA4-1						75	71	78	71	-	75	71	78	71	-	-
pAhHMA4-2											100	100	98	97	-	95

Fig. S3. Sequence conservation in *HMA4* promoters of *A. thaliana* and *A. halleri*. Schematic representation of *pAhHMA4-2* and *pAhHMA4-3* as described in this study as well as *pAhHMA4-1* and *pAtHMA4* described previously (Hanikenne *et al.*, 2008). Pairwise comparisons using BLASTN (NCBI) allowed identification of different regions sharing more than 71% identity between at least two promoters (colored blocks). The 5'UTR and the intron located upstream the ATG start codon (*) are represented by solid and dotted lines, respectively. The thick black lines represent the sequences cloned in Hanikenne *et al.* (2008). The table shows the percentage of identity between each region.

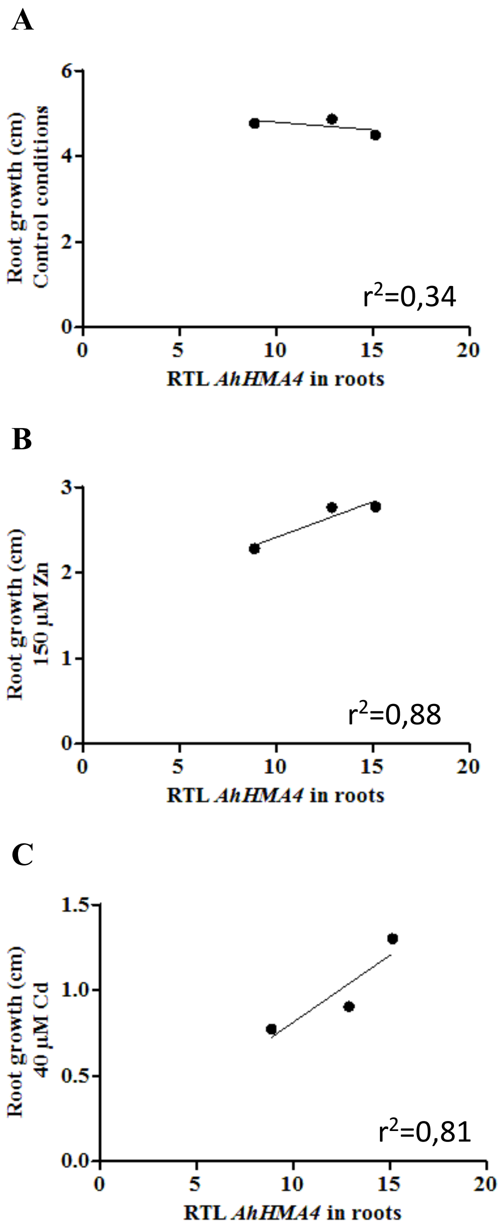

Fig. S4. Relationship between root metal tolerance and *AhHMA4* transcript levels. Root growth (cm) in control conditions (1 µM Zn) (A), 150 µM Zn (B) or 40 µM Cd (C) were plotted against Relative Transcript Level (RTL) of *AhHMA4* in roots. Technical details are provided in Figs 2 and 5. The dots correspond to mean values in transgenic homozygous plants expressing *AhHMA4::GFP* under the control of *pAhHMA4-1*, *pAhHMA4-2* and *pAhHMA4-3* with *pAhHMA4-1* having the lowest *AhMHA4* RTL and *pAhHMA4-3* having the highest *AhMHA4* RTL. Linear regression and coefficient of correlation (r^2) is plotted for each graph.

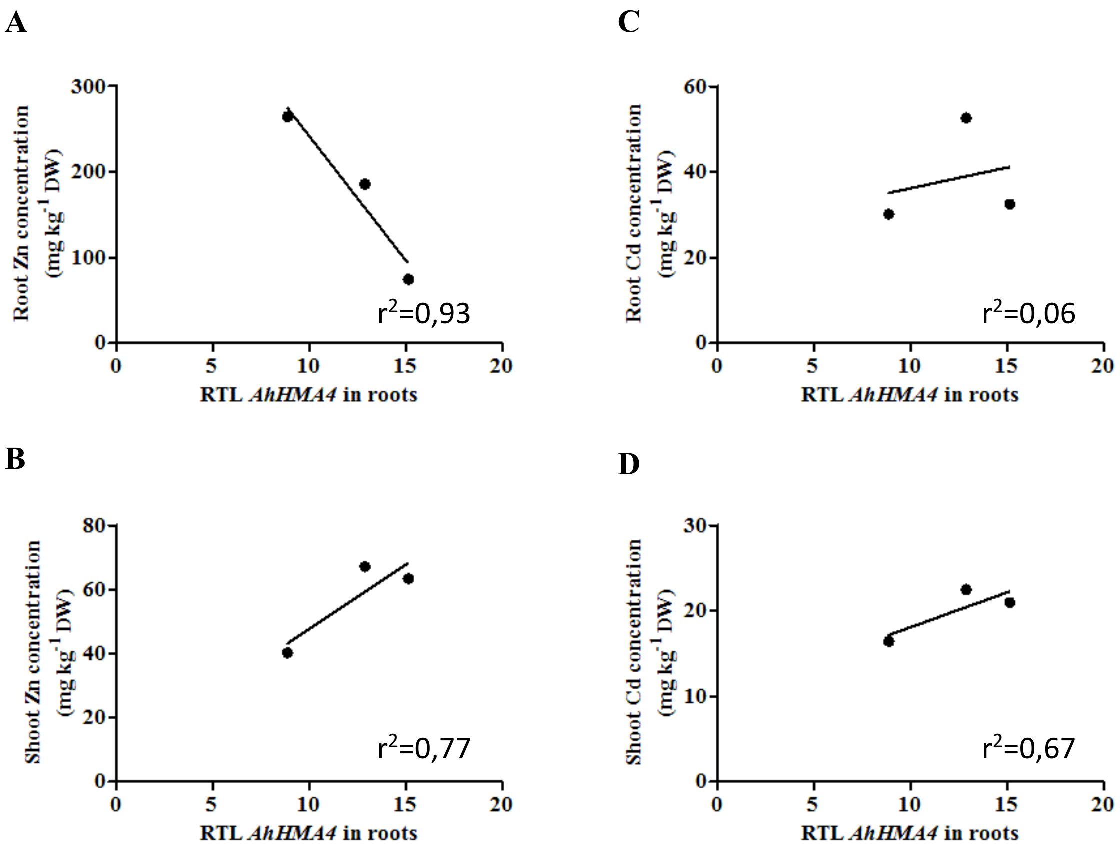

Fig. S5. Relationship between Zn or Cd accumulation and *AhHMA4* transcript levels. Zn and Cd accumulation in roots (A, C) and shoots (B, D) of plants grown at 0.2 µM Zn (A, B) or 0.05 µM Cd (C, D), respectively, were plotted against Relative Transcript Level (RTL) of *AhHMA4* in roots. Technical details are provided in Figs 2 and 6. The dots correspond to mean values in transgenic homozygous plants expressing *AhHMA4::GFP* under the control of *pAhHMA4-1*, *pAhHMA4-2* and *pAhHMA4-3* with *pAhHMA4-1* having the lowest *AhMHA4* RTL and *pAhHMA4-3* having the highest *AhMHA4* RTL. Linear regression and coefficient of correlation (r^2) is plotted for each graph.

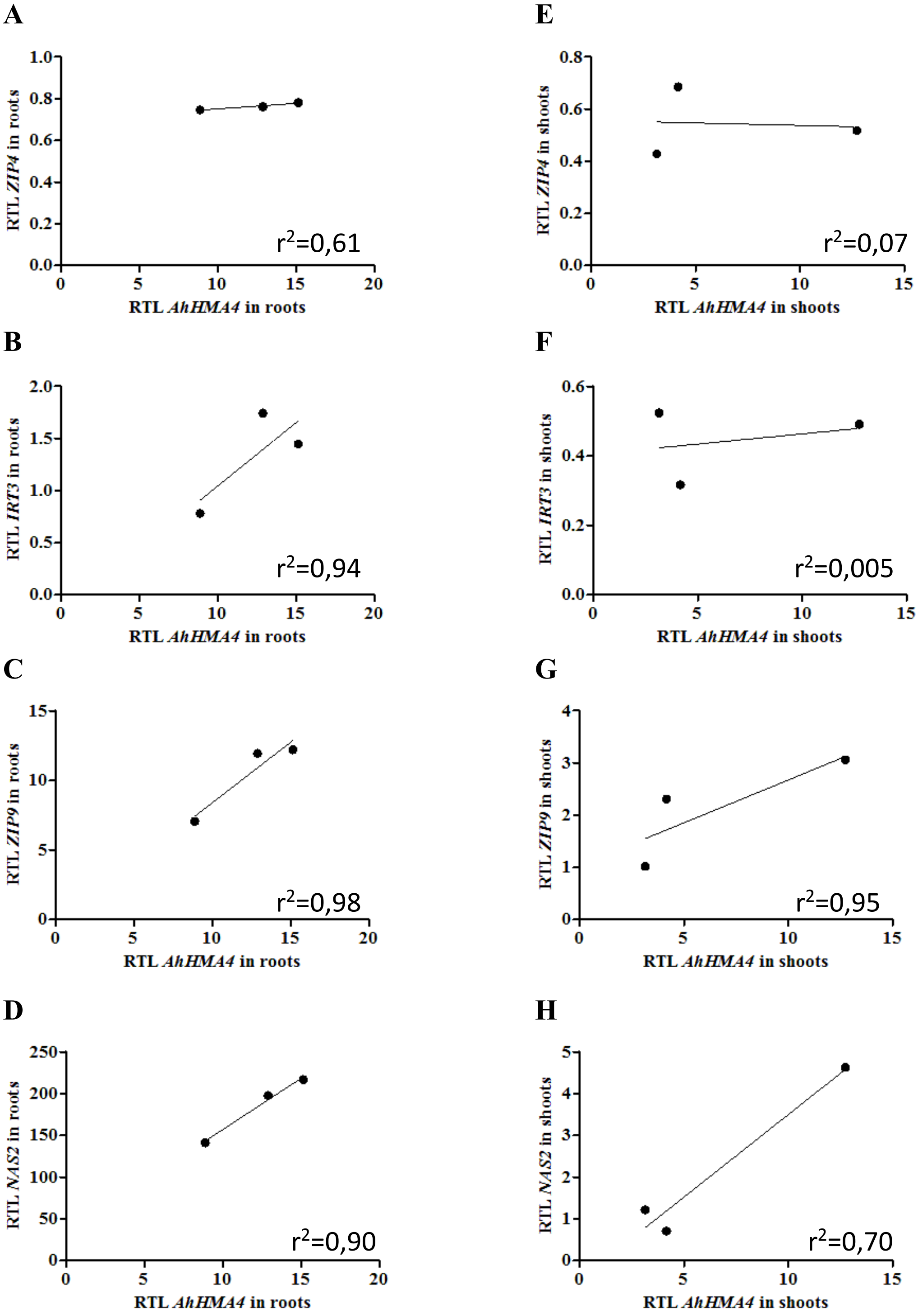

Fig. S6. Relationship between expression of Zn-responsive genes and *AhHMA4*. The Relative Transcript Level (RTL) of Zn-responsive genes *IRT3* (A, E), *ZIP4* (B, F), *ZIP9* (C, G) and *NAS2* (D, H) in roots (A-D) and shoots (E-H) of plants grown at 0.2 µM Zn were plotted against *AhHMA4* RTL in roots (A-D) or shoots (E-H). Technical details are provided in Figs 2, 7 and 8. The dots correspond to mean values in transgenic homozygous plants expressing *AhHMA4::GFP* under the control of *pAhHMA4-1*, *pAhHMA4-2* and *pAhHMA4-3* with *pAhHMA4-1* having the lowest *AhMHA4* RTL and *pAhHMA4-3* having the highest *AhMHA4* RTL. Linear regression and coefficient of correlation (r^2) is plotted for each graph.

Table S1. Sequences and reaction efficiencies of primer pairs used for real-time RT-PCR.

Gene	Species	Forward 5'-3'	Reverse 5'-3'	Reaction efficiency	Standard deviation RE
EF1a	***A. thaliana***	TGAGCACGCTCTTCTTGCTTTCA	GGTGGTGGCATCCATCTTGTTACA	1.983	0.051
UBQ10	***A. thaliana***	GGCCTTGTATAATCCCTGATGAATAAG	AAAGAGATAACAGGAACGGAAACATAGT	2.046	0.052
ZIP3	***A. thaliana***	GGAGTTTTGAACGCTGCATCC	CTGCGAGAAAGTCCACCAGAGA	1.943	0.081
ZIP4	***A. thaliana***	AGCAAGAGAGGAATCAGGCTGC	CCAACCACGGGAACAACAGCA	1.913	0.047
ZIP9	***A. thaliana***	CCATCACTACTCCCATCGGTGT	CACCAATGACGCAACGCTATAA	1.858	0.051
IRT3	***A. thaliana***	AGTCACCCTCCTGGTCATGGTT	GCCCATGCCCAATGTCAAT	1.951	0.058
NAS2	***A. thaliana***	CCGATGATGTGGTTAATTCGG	TGCCTCGAGCTCCATTTGA	1.946	0.088
HMA4	***A. thaliana***	AGCTAGCTACAAGGCGACCTCG	CAGCTTTAACTGCTACAACTGTATC	1.932	0.083
HMA4	***A. halleri***	AGCTAGCTACAGGGCGACAGCA	CAGCTTTAACCGCTACAACTGTGCT	1.908	0.062

RE = reaction efficiency
